# Supplementary material for: Risk Factors for Anemia and Micronutrient Deficiencies among Women of Reproductive Age—The Impact of the Wheat Flour Fortification Program in Uzbekistan
Source: Nutrients. 2020 Mar 7;12(3):714. doi: 10.3390/nu12030714 (PMC7146397; doi:10.3390/nu12030714)
Supplement: Supplementary file 1 [file nutrients-12-00714-s001.pdf]

Supplementary table 1. Results of bivariate analysis of associations of potential risk factors of anemia, iron and folate deficiency, non-pregnant women, Uzbekistan 2017

		Anemia			Iron deficiency			Folate deficiency		
**Characteristics**	**Value**	**Number with anemia**	**% [a]**	**P value [b]**	**Number with iron deficiency**	**% [a]**	**P value [b]**	**Number with folate deficiency**	**% [a]**	**P value [b]**
Demographics										
Residence	Urban	85	17.8%	0.16	237	51.7%	0.10	234	49.3%	0.09
	Rural	275	21.7%		562	46.9%		527	43.9%	
Region	Andijon	26	26.3%	<0.001	40	42.1%	<0.05	59	61.5%	<0.001
	Bukhara	22	15.8%		56	42.4%		70	52.2%	
	Ferghana	27	21.6%		55	45.8%		69	56.6%	
	Jizzakh	17	15.5%		42	40.8%		52	50.5%	
	Karakalpakstan	52	36.9%		85	62.0%		75	54.3%	
	Kashqadarya	16	13.0%		54	45.4%		55	45.5%	
	Khorazm	35	23.2%		51	41.5%		26	21.1%	
	Namangan	13	11.0%		56	47.5%		66	55.9%	
	Navoiy	17	15.5%		58	54.7%		44	41.5%	
	Samarkand	13	11.6%		48	45.7%		33	31.4%	
	Surkhandarya	26	16.0%		67	45.6%		68	46.3%	
	Sirdarya	23	19.0%		55	48.2%		51	44.7%	
	Tashkent city	22	18.3%		68	58.6%		48	40.7%	
	Tashkent oblast	51	41.8%		64	54.7%		45	38.1%	
Households wealth quintile	Poorest	66	19.9%	<0.05	136	42.9%	0.15	138	44.9%	0.72
	Second	64	18.1%		163	49.0%		150	45.0%	
	Middle	83	22.4%		159	45.9%		145	42.6%	
	Fourth	82	26.3%		154	52.0%		140	45.3%	
	Wealthiest	63	16.3%		184	52.1%		183	48.8%	
Woman's household had adequate sanitation	Yes	330	21.2%	0.54	720	49.3%	0.023	669	45.3%	0.80
	No	30	15.5%		79	40.2%		92	46.5%	
Woman's household had access to safe water source	Yes	346	20.7%	0.78	766	48.5%	0.993	719	44.9%	0.12
	No	14	19.2%		33	48.6%		37	56.3%	
Woman's education	Secondary or less	158	21.2%	0.62	314	44.1%	<0.01	329	46.0%	0.73
	Special secondary or higher	202	20.1%		485	51.4%		432	45.1%	
Woman's age (years)	15-19	35	17.8%	0.05	91	49.8%	0.53	78	40.5%	<0.05

Characteristics	Value	Anemia: Number with anemia	Anemia: % [a]	Anemia: P value [b]	Iron deficiency: Number with iron deficiency	Iron deficiency: % [a]	Iron deficiency: P value [b]	Folate deficiency: Number with folate deficiency	Folate deficiency: % [a]	Folate deficiency: P value [b]
	20-24	45	15.3%		128	46.3%		118	43.2%	
	25-29	76	23.7%		171	54.0%		171	53.1%	
	30-34	61	18.1%		136	47.2%		113	40.4%	
	35-39	53	25.7%		94	47.1%		99	48.9%	
	40-44	38	21.3%		77	45.2%		88	50.4%	
	45-49	52	23.0%		102	46.6%		94	40.5%	
Woman's physiology and nutrition										
Woman currently lactating	Yes	72	20.5%	0.97	168	54.1%	0.03	172	54.8%	<0.001
	No	288	20.6%		631	47.0%		589	43.2%	
Woman smokes cigarettes	Yes	2	25.2%	0.76	3	29.7%	0.35	6	65.1%	0.34
	No	358	20.5%		796	48.4%		755	45.3%	
Woman is underweight	Yes	17	14.8%	0.14	41	39.3%	0.08	47	45.4%	0.99
	No	343	20.9%		756	48.8%		714	45.5%	
Woman is overweight/obese	Yes	153	20.6%	0.99	341	48.3%	0.96	334	47.5%	0.18
	No	207	20.6%		456	48.2%		427	44.1%	
Woman took iron supplements in past 6 months	Yes	39	21.7%	0.74	72	42.4%	0.10	81	48.3%	0.53
	No	319	20.5%		718	49.1%		674	45.2%	
Woman took folic acid supplements in past 6 months	Yes	9	19.7%	0.86	18	41.8%	0.37	28	60.4%	0.06
	No	350	20.7%		775	48.7%		729	45.2%	
Woman had minimal dietary diversity past day	Yes	142	20.2%	0.94	335	49.8%	0.31	322	48.1%	0.17
	No	199	20.3%		436	47.1%		418	44.3%	
Woman consumed iron-rich foods past day [c]	Yes	74	18.9%	0.44	175	46.3%	0.44	160	42.0%	0.12
	No	279	20.8%		613	48.8%		592	46.5%	
Woman consumed folate-rich foods past day [d]	Yes	203	19.7%	0.43	464	48.3%	0.90	439	44.6%	0.40
	No	145	21.5%		314	47.9%		305	46.9%	
Iron intake from fortified flour (as % RNI)	0%	228	19.5%	0.22	541	49.6%	0.40	544	49.2%	<0.001
	>0 – 39.9%	63	21.8%		128	44.6%		101	34.2%	
	40+%	48	24.8%		84	48.2%		59	31.1%	
Folate intake from Fortified flour (as % RNI)	0%	228	19.5%	0.27	541	49.6%	0.41	544	49.2%	<0.001
	>0 – 69.9%	61	22.2%		121	44.6%		97	34.7%	
	70+%	50	24.0%		91	48.0%		63	30.7%	
Woman micro-nutrition and inflammation										
Woman has inflammation	Yes	53	17.4%	0.20	123	43.4%	0.10	149	53.7%	<0.01

Characteristics	Value	Anemia: Number with anemia	Anemia: % [a]	Anemia: P value [b]	Iron deficiency: Number with iron deficiency	Iron deficiency: % [a]	Iron deficiency: P value [b]	Folate deficiency: Number with folate deficiency	Folate deficiency: % [a]	Folate deficiency: P value [b]
	No	273	20.9%		676	49.4%		605	43.5%	
Woman is iron deficient	Yes	62	33.3%	<0.001				373	46.7%	0.31
	No	273	7.9%					381	44.0%	
Woman is vitamin A	Yes	144	30.3%		283	61.8%	0.31	203	43.5%	0.44
insufficient	No	180	16.1%	<0.001	480	42.8%		512	45.9%	
Woman is folate	Yes	167	21.6%	0.22	373	49.8%	0.82			
deficient	No	170	19.0%		426	47.1%				
Woman is vitamin B12	Yes	65	19.9%	0.89	154	47.7%	0.10	138	41.9%	0.20
deficient	No	272	20.2%		645	48.5%		623	46.3%	

Note: The n’s are un-weighted numbers for each subgroup.

[a] Percentages or means are weighted for unequal probability of selection, except distribution of regions.

[b] Chi-square test; P values <0.1 were retained in the multivariable model, except for iron and folate intakes (as % RNI) which were forced into the model regardless of the results of the bivariate analysis.

[c] The following food groups were included here: a) liver, kidney, heart, or other organ meats; b) any meat, such as beef, lamb, goat, chicken, rabbit or duck.

[d] The following food groups were included here: a) nuts and seeds; b) any dark green leafy vegetables; c) any foods made from beans, peas, lentils; d) liver, kidney, heart, or other organ meats.
